# Dynamic response and liquefaction potential of porous seabed induced by partial standing ocean waves

**DOI:** 10.1038/s41598-023-45485-6

**Published:** 2023-11-04

**Authors:** Guocai Wang, Yiyang Liu, Kai Liu, Chate Xu

**Affiliations:** 1 Zhejiang Key Laboratory of Civil Engineering Structures & Disaster Prevention and Mitigation Technology, Zhejiang University of Technology, Hangzhou, 310023 China; 2 College of Civil Engineering, Zhejiang University of Technology, Hangzhou, 310023 China; 3 Department of Civil and Environmental Engineering, Louisiana State University, Baton Rouge, LA 70803 USA

**Keywords:** Civil engineering, Ocean sciences

## Abstract

The analysis of ocean wave-induced dynamic response of a porous seabed is particularly important for coastal and geotechnical engineers when designing and constructing maritime structures. In this study, an analytical solution is presented to analyze the dynamic response and liquefaction potential of a poro-elastic seabed induced by partial standing waves with arbitrary reflectivity. The porous seabed is modeled using Biot’s theory describing the propagation of elastic waves, and coupled deformation and water flow of porous media, whereas the ocean waves are described using linear ocean wave theory. Based on the mixed boundary-value conditions, explicit expressions of displacements, effective stresses and excess pore water pressure of seabed are derived with consideration of the effects of inertial forces, compressibility of solid and fluid, and arbitrary reflectivity of standing waves. The results of degenerated analytical solutions are compared with the existing ones to verify the correctness of the proposed method. The effects of several pertinent parameters of ocean wave-seabed system, including reflection coefficient, phase lag and period of standing waves, depth of water, permeability, degree of saturation, and shear modulus of seabed deposits, etc., on the dynamic response of seabed and liquefaction potential, are examined and discussed. It is found that the reflection of standing wave has a significant effect on the dynamic response and liquefaction potential of porous elastic seabed. Compared with that of no wave reflection, the liquefaction depth of seabed induced by fully-reflected standing waves increases 82.49% under certain conditions of wave-seabed system. In addition, phase lag, wave period, water depth and mechanical and physical properties of seabed soil such as saturation, permeability and shear modulus have different effects on the dynamic response and liquefaction potential of porous elastic seabed. The investigation of the dynamic response and liquefaction of the porous elastic seabed under partial standing ocean waves will help to better predict the influence of standing waves on breakwaters and seabed soil, and can provide some guidance for the design of offshore structures.

## Introduction

When progressive ocean waves arrive normally at maritime structures, such as wharf, breakwaters, offshore oil platform, seawalls, caisson structures, piers, levees, etc., they may be reflected partially or fully from it, resulting in standing waves. According to the theories of linear water waves, the height of resultant waves may reach twofold of its incident component for fully reflected cases. Compared with that of progressive water waves, a stronger wave impact on the maritime structures will be produced, accompanied by complex interactions between water and soil particles, which will enhance the scour and possibility of slope failure of sedimentary bed^1–4^. Therefore, it is necessary to evaluate the variations of stresses, pore water pressure and liquefaction susceptibility of marine sediments induced by ocean waves in detail when designing and constructing offshore structures.

Numerous investigations focusing on the dynamic interactions among waves, seabed and maritime structures have been made since the 1970s. Different analysis methods, such as analytical, numerical or experimental methods, especially analytical ones, were used in the investigation. A number of diverse theories have been proposed analytically to study the distribution of pore water pressure, stresses and liquefaction depth in isotropic or cross-anisotropic seabed induced by progressive or standing waves, and the effects of properties of ocean wave and seabed soil on the dynamic response of poroelastic seabed were investigated and discussed^5–10^. Besides analytical methods, numerical simulation is also a useful tool to investigate the dynamic interactions among ocean waves, offshore structures and seabed deposits. Different water waves, complex constitutive relations of seabed soil, and irregular boundaries of the topics can be modeled^11–18^.

However, most of the above investigations considered the behavior of porous seabed governed by consolidation theory^19^ and storage equation^20^. In the analysis, the effects of both inertia terms of soil and of fluid on the dynamic response of seabed were ignored, which might give inaccurate prediction under certain conditions of wave-seabed system^2,21–25^. Considering the influence of inertia terms of both fluid and solid on the dynamic response of ocean sediments, Jeng et al. discussed the applicable range of fully coupled dynamic model and quasi-static approximations^22^. Lin and Li considered the effects of Coulomb friction and investigated the instability of seabed caused by ocean waves^26^. Kumagai and Foda established an analytical model to evaluate the dynamic response of porous seabed beneath composite breakwater^23^. Ulker et al. and Ye et al. used finite element algorithm and studied the dynamic response and instability of ocean sediment around breakwaters subjected to breaking or standing waves^2,21,27^. Recently, Zhang et al. proposed a three-dimensional poro-elasto-plastic model to analyze the response of seabed around pipelines induced by water waves, where the influence of porosity and plasticity properties of soil were examined^28^. Yang and Ye investigated the residual liquefaction susceptibility induced by standing waves in a loosely ocean sediment using validated integrated numerical model^4^. Wang et al. proposed an analytical method to reveal the dynamic response and liquefaction of a poroelastic seabed subjected to progressive ocean waves using Biot’s fully coupled theory^24,25^. Considering the obstacles that may be encountered in the process of water wave transmission, Barman and Bora studied the influence of the interaction between the porous elastic structure near the partially reflected seawall and the oblique water wave in the two-layer fluid flowing on the porous seabed^29–32^. Based on the linear wave theory, Mohapatra and Guo established an analytical model for the interaction between oblique waves and breakwaters composed of underwater horizontal flexible porous membranes near vertical porous barriers^33–36^.

It can be seen from the above studies that the understanding and predicting the dynamic response and liquefaction of the porous elastic seabed are crucial for the safe and efficient operation of offshore infrastructure. To the best of the authors’ knowledge, the analytical solutions of the dynamic response of seabed induced by partial standing waves, whereas the effects of inertia terms and the coupling of solid and fluid, and the reflection of incident waves were considered, were not reported in the literature. In the offshore environment, the particle arrangement of seabed soil is generally loose. Under the action of periodic wave loads (especially the synthetic waves generated by standing waves), the wave-induced periodic shear stress ratio at a certain position of the seabed is greater than the critical value, causing the seabed soil to undergo shrinkage plastic deformation. Due to the characteristics of seabed soil, part of the pore water in the seabed is discharged through the seabed surface driven by the upward pore pressure gradient, and the soil particles will rearrange their relative positions to make them denser. In this process, the pore water pressure increases, causing the soil to liquefy or soften. Therefore, the construction of marine structures in the offshore environment will bring huge risks. Therefore, it is of great significance to analyze the dynamic response of porous elastic seabed under partial standing waves with arbitrary reflectivity.

Through the above analysis, we realize the importance of investigating the dynamic response of the seabed caused by partial standing waves. The main purpose of this study is to propose an analytical solution to analyze the dynamic response of the porous elastic seabed under partially standing waves with arbitrary reflectivity. The new contributions of this paper are summarized as follows.

1. Based on the linear water wave theory, the fluid pressure caused by partial standing waves is obtained. Combined with Biot theory, effect stress principle and generalized Hooke’s law, the dynamic response and liquefaction potential of poroelastic seabed induced by partial standing waves can be evaluated, including excess pore pressure, effective normal stress and shear stress, and the liquefaction depth of seabed soil.
2. The interaction between the standing wave and the seabed soil is analyzed. The effects of different wave reflection coefficients, phase lag, wave period, water depth, fluid compressibility, shear modulus and soil permeability on the effective vertical stress and excess pore pressure of the coastal bottom depth, and the design of the breakwater foundation, are discussed.
3. The influence of standing wave on the liquefaction depth of porous seabed in different wave periods is discussed, and the change of liquefaction depth is explained.

The structure of this paper is as follows: Firstly, considering the influence of inertial force, the compressibility of soil and fluid, and the arbitrary reflectivity and phase lag of some standing waves, the explicit solution is derived. Then, the results of the degradation analytical solution are compared with the results of the existing analytical solutions to verify the validity and correctness of the proposed theory. Subsequently, some selected numerical results are given at the end of the paper. The effects of seabed soil and wave properties on the dynamic response and liquefaction potential of the wave-seabed system are discussed. Finally, the main conclusions of this study are summarized.

## Mathematical formulation

In the analysis, the fluid pressure $p\left(x,z,t\right)$ induced by partial standing waves can be firstly derived using the theories of linear iteration and linear water wave. Then, supposing that a harmonic loading (e.g., bottom pressure of water waves) is applied on the surface of the porous seabed, the general solutions of displacements and stresses of the media can be obtained. Finally, based on the mixed boundary-value conditions, the explicit expressions of displacements, effective stresses and excess pore water pressure of seabed under arbitrary partial standing waves can be derived.

### Dynamic fluid pressure under partial standing waves

**Figure 1:**
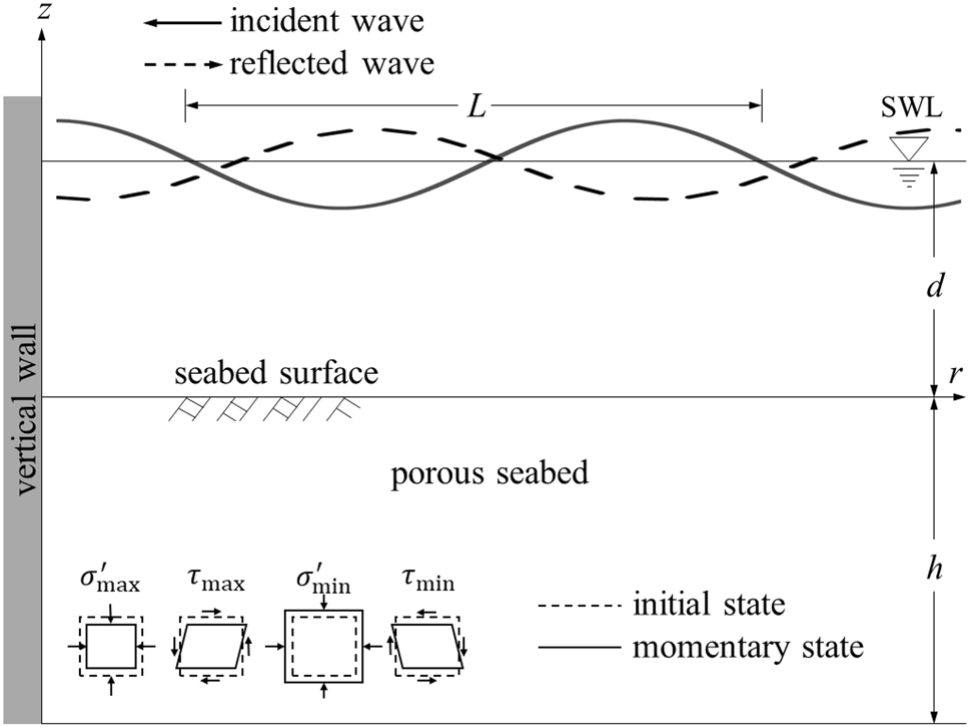
Interactions between standing ocean waves and porous seabed.

When arriving normally at maritime structures, incident waves may be reflected to form partially standing waves (Fig. 1). A large wave impact will be applied on the maritime structures, causing complex motions between water and seabed soils, which will enhance the scour of seabed and the failure of submarine slope. According to the theory of linear water waves, the profile of total sea surface elevation $\eta \left(x,t\right)$ can be expressed as

1 $$\eta \left(x,t\right)=\frac{{H}_{i}}{2}\mathrm{cos}\left(kx-\omega t\right)+\frac{{H}_{r}}{2}\mathrm{cos}\left(kx+\omega t+\epsilon \right),$$

where ${H}_{i}$ and ${H}_{r}$ = heights of incident and reflected waves, respectively; $k$ = wave number, $k=2\pi /L$; $\omega$ = radian frequency, $\omega =2\pi /T$; $L$ = wave length; $T$ = wave period; $\epsilon$ = phase lag of reflection waves; and $t$ = time.

For partial standing waves, the associated velocity potential $\phi \left(x,z,t\right)$ can be written as^37^

2 $$\phi \left(x,z,t\right)=-\frac{{H}_{i}L}{2T}\frac{\mathrm{cosh}\left(kz\right)}{\mathrm{sinh}\left(kd\right)}\left[\mathrm{sin}\left(kx-\omega t\right)-{K}_{R}\mathrm{sin}\left(kx+\omega t+\epsilon \right)\right],$$

in which ${K}_{R}$ = reflection coefficient of reflection waves, ${K}_{R}={H}_{r}/{H}_{i}$.

The dispersion relationship for small-amplitude waves in finite depth of water (i.e., $0.05<d/L<0.5$) can be written as

3 $${\omega }^{2}=gk\mathrm{tanh}\left(kd\right),$$

where $d$ = depth of water, and $g$ = gravitational acceleration.

From Eq. (3), it can be seen that the wave length is determined by period of wave and depth of water. For different values of $d$ and $\omega$, the value of $k$ is different. Furthermore, Eq. (3) is a nonlinear equation, whose root can be obtained numerically using numerical calculation methods, such as bisection method, iterative method, Newton’s tangential method, etc. In this paper, we use the iterative method to obtain the wave length for given values of depth of water and period of wave.

According to the linear wave theory^37^, the unsteady Bernoulli equation for irrotational and incompressible fluid can be written as

4 $$-\frac{\partial \phi }{\partial t}+\frac{1}{2}\left[{\left(\frac{\partial \phi }{\partial x}\right)}^{2}+{\left(\frac{\partial \phi }{\partial z}\right)}^{2}\right]+\frac{p}{{\rho }_{w}}+g(z-d)=0,$$

in which $p$ = fluid pressure; ${\rho }_{w}$ = bulk density of water.

Therefore, the fluid pressure $p\left(x,z,t\right)$ induced by partial standing waves can be obtained by substitution of Eq. (2) into Eq. (4), rendering

5 $$p\left(x,z,t\right)=-{\rho }_{w}g\left(z-d\right)+\frac{{\gamma }_{w}{H}_{i}}{2}\frac{\mathrm{cosh}\left(kz\right)}{\mathrm{cosh}\left(kd\right)}\left[\mathrm{cos}\left(kx-\omega t\right)+{K}_{R}\mathrm{cos}\left(kx+\omega t+\epsilon \right)\right].$$

### Governing equations for porous seabed

In 1956, Biot established a general theory describing the dynamic behavior of a poroelastic, saturated/partially saturated medium, where fluid flow in a porous medium is characterized by the conventional Darcy’s law, and the solid frame is taken to be isotropic and elastic. Therefore, omitting body forces, the dynamic equilibrium equations for isotropic, poro-elastic soil and fluid can be expressed as^38^

6 $${\sigma }_{ij,j}=\rho {\ddot{u}}_{i}+{\rho }_{w}{\ddot{w}}_{i},$$

7 $$-{p}_{f,i}={\rho }_{w}{\ddot{u}}_{i}+\frac{{\rho }_{w}}{n}{\ddot{w}}_{i}+\frac{{\gamma }_{w}}{{k}_{c}}{\dot{w}}_{i},$$

where ${\sigma }_{ij}$ ($i,j=x,y,z$) = total stress tensor; ${p}_{f}$ = excess pore pressure; ${u}_{i}$ and ${w}_{i}$ = displacement components of solid and of fluid relative to soil skeleton, respectively; $\rho$ is combined bulk density of soil, $\rho =n{S}_{r}{\rho }_{w}+(1-n){\rho }_{s}$ with $n$ = porosity, ${S}_{r}$ = saturation degree of soil, and ${\rho }_{s}$ = bulk density of solid skeleton; ${k}_{c}$ = coefficient of permeability. A dot above a variable indicates differentiation with respect to time $t$, while a comma in the subscript denotes a partial derivative with respect to spatial coordinate.

The continuity equation associated with the fluid flow in a porous soil can be expressed as^19^

8 $${\dot{p}}_{f}+\alpha M{\dot{\varepsilon }}_{ii}+M{\dot{w}}_{i,i}=0,$$

where ${\varepsilon }_{ii}$ = volumetric strain of solid; $\alpha$ = Biot coefficient of effective stress, and $M$ = Biot parameters^39^. The parameters $\alpha$ and $M$ can be defined alternatively as

9 $$\alpha =1-\frac{K}{{K}_{s}}, M=\frac{{K}_{f}{K}_{s}^{2}}{{K}_{f}\left({K}_{s}-K\right)+n{K}_{s}\left({K}_{s}-{K}_{f}\right)},$$

where $K$ and ${K}_{s}$ = bulk modulus of soil and of solid frame, respectively; ${K}_{f}=({\gamma }_{w}d{K}_{w})/[{\gamma }_{w}d+{K}_{w}\left(1-{S}_{r}\right)]$, with ${K}_{w}$ = bulk modulus of air-free fluid.

According to the principle of effective stresses, the relations between effective and total stresses can be written as

10 $${\sigma }_{ij}={\sigma }_{ij}{\prime}-\alpha {p}_{f}{\delta }_{ij},$$

where ${\delta }_{ij}$ = Kronecker delta function; ${\sigma }_{ij}{\prime}$ = effective stress tensor of solid skeleton.

According to the generalized Hooke’s law, the effective stress–strain relations for solid skeleton are defined as

11 $${\sigma }_{ij}{\prime}=\lambda {\varepsilon }_{kk}{\delta }_{ij}+2G{\varepsilon }_{ij},$$

where $\lambda$ = Lamé constant, $G$ = shear modulus of soil. ${\varepsilon }_{ij}$ is the strain of soil skeleton defined as

12 $${\varepsilon }_{ij}=\frac{1}{2}\left({u}_{i,j}+{u}_{j,i}\right).$$

The governing Eqs. (6) to (8) are characterized by three independent Rice–Cleary micromechanical parameters, $K$, ${K}_{s}$, and ${K}_{f}$, together with porosity $n$, coefficient of permeability ${k}_{c}$, mass density of solid ${\rho }_{s}$ and of fluid ${\rho }_{w}$. The expressions of $\alpha$ and $M$ can be simplified to simpler forms for some special cases. For examples, for ideal porous medium, if the soil skeleton is incompressible, i.e., $K/{K}_{s}\ll 1$, it renders $\alpha =1$, and $M={K}_{f}/n$; if both the seabed soil and pore fluid are incompressible, i.e., $K/{K}_{s}\ll 1$ and ${K}_{f}\to \infty$, it yields $\alpha =1$, and $M\to \infty$; if only the pore fluid within the seabed soil is incompressible, i.e., ${K}_{f}\to \infty$, it renders $\alpha =1-K/{K}_{s}$, and $M={K}_{s}^{2}/[\left(1-n\right){K}_{s}-K]$; and if the pore fluid within the seabed is infinitely compressible i.e., ${K}_{f}=0$, it yields $\alpha =1-K/{K}_{s}$, and $M=0$.

### Boundary conditions and general solutions

In principle, it can be approximately considered that the shear stress is related to the oscillating flow above the seabed surface. However, in the actual calculation, the fluid shear stress and the vertical effective normal stress on the seabed surface are very small and can be ignored^9^. Since our governing equations are valid only for materials finer than gravel, we may therefore evaluate the pressure exerted at the surface of the porous seabed by the waves from a wave theory which assumes the bottom to be impermeable. For simplicity we choose to describe the wave motion by linear wave theory^5^. For constant water depth $d$, we have the wave associated bottom pressure ${p}_{0}$. In terms of complex variables the boundary condition imposed by the wave motion at the surface of the porous seabed is

13 $${\sigma }_{z}{\prime}={\tau }_{xz}=0, {p}_{f}={p}_{b}=-{p}_{0}\left[\mathrm{cos}\left(kx-\omega t\right)+{K}_{R}\mathrm{cos}\left(kx+\omega t+\epsilon \right)\right],$$

where ${p}_{b}$ = bottom pressure of ocean waves; ${p}_{0}={\gamma }_{w}{H}_{i}/[2\mathrm{cosh}\left(kd\right)]$.

For a poroelastic seabed with a finite thickness $h$ (Fig. 1), the underlying soil is assumed to be rigid and impermeable, and the displacement component of the solid and excess pore water pressure gradient at $z=-h$ is zero. Therefore, the boundary condition at the seabed $z=-h$ can be described as (Lin and Li^26^; Zhang et al.^28^)

14 $${u}_{x}={u}_{z}=\partial {p}_{f}/\partial z=0,$$

If the thickness of seabed is infinite, the displacements and excess pore pressure must be zero to assure radiation condition as $z$ approaches negative infinity. Therefore, the boundary conditions at $z\to -\infty$ can be written as

15 $${u}_{x}={u}_{z}={p}_{f}=0.$$

By virtue of Euler formula, the general solutions of ordinary differential Eqs. (6) to (8) can be written as follows

16 $${u}_{x}=\sum_{j=1}^{6}{A}_{j}{e}^{{\eta }_{j}z}\left\{\left[\mathrm{cos}kx-{K}_{R}\mathrm{cos}\left(kx+\epsilon \right)\right]+i\left[\mathrm{sin}kx+{K}_{R}\mathrm{sin}\left(kx+\epsilon \right)\right]\right\}{e}^{-i\omega t},$$

17 $${u}_{z}=\sum_{j=1}^{6}{B}_{j}{A}_{j}{e}^{{\eta }_{j}z}\left\{\left[\mathrm{cos}kx+{K}_{R}\mathrm{cos}\left(kx+\epsilon \right)\right]+i\left[\mathrm{sin}kx-{K}_{R}\mathrm{sin}\left(kx+\epsilon \right)\right]\right\}{e}^{-i\omega t},$$

18 $${p}_{f}=\sum_{j=1}^{6}{C}_{j}{A}_{j}{e}^{{\eta }_{j}z}\left\{\left[\mathrm{cos}kx+{K}_{R}\mathrm{cos}\left(kx+\epsilon \right)\right]+i\left[\mathrm{sin}kx-{K}_{R}\mathrm{sin}\left(kx+\epsilon \right)\right]\right\}{e}^{-i\omega t},$$

where ${\eta }_{1,2}=\pm \sqrt{{k}^{2}-{k}_{f}^{2}}$, ${\eta }_{3,4}=\pm \sqrt{{k}^{2}-{k}_{s}^{2}}$, and ${\eta }_{5,6}=\pm \sqrt{{k}^{2}-{k}_{t}^{2}}$; ${k}_{f}^{2}=({\beta }_{1}+\sqrt{{\beta }_{1}^{2}-4{\beta }_{2}})/2$, ${k}_{s}^{2}=({\beta }_{1}-\sqrt{{\beta }_{1}^{2}-4{\beta }_{2}})/2$, and ${k}_{t}^{2}={\alpha }_{3}/G$; ${\alpha }_{1}={\rho }_{w}{\omega }^{2}/n+i\omega {\gamma }_{w}/{k}_{c}$, ${\alpha }_{2}=\alpha -{\rho }_{w}{\omega }^{2}/{\alpha }_{1}$, ${\alpha }_{3}=\rho {\omega }^{2}-{\rho }_{w}^{2}{\omega }^{4}/{\alpha }_{1}$, ${\alpha }_{4}={\rho }_{w}{\omega }^{2}-\alpha {\alpha }_{1}$, and ${\alpha }_{5}={\alpha }_{1}/M$; ${\beta }_{1}={\alpha }_{5}+({\alpha }_{3}-{\alpha }_{2}{\alpha }_{4})/(\lambda +2G)$, and ${\beta }_{2}={\alpha }_{3}{\alpha }_{5}/(\lambda +2G)$.

The coefficients of ${B}_{j}$ and ${C}_{j}$ ($j=\mathrm{1,2},\dots ,6$) appeared in Eqs. (17) and (18) are

19 $${B}_{j}=\left\{\begin{array}{l}-i\frac{{\eta }_{j}}{k}, \quad  j=\mathrm{1,2},\mathrm{3,4}\\ -i\frac{k}{{\eta }_{j}},  \quad j=\mathrm{5,6}\end{array}\right.,$$

20 $${C}_{j}=\left\{\begin{array}{l}i\frac{{\alpha }_{1}{\alpha }_{3}-{\alpha }_{1}(\lambda +2G){k}_{f}^{2}}{k{\alpha }_{4}}, \quad j=\mathrm{1,2}\\ i\frac{{\alpha }_{1}{\alpha }_{3}-{\alpha }_{1}(\lambda +2G){k}_{s}^{2}}{k{\alpha }_{4}}, \quad j=\mathrm{3,4}\\ 0 ,j=\mathrm{5,6}\end{array}\right..$$

The values of ${A}_{j}$ ($j=\mathrm{1,2},3,\dots ,6$) appeared in Eqs. (16) to (18) can be obtained from appropriate boundary conditions. For the seabed of finite thickness, it yields from Eqs. (13) and (14) that

21 $${A}_{j}={{\varvec{R}}}^{-1}\cdot {\varvec{B}},$$

where ${\varvec{B}}=\{\mathrm{0,0},\mathrm{0,0},0,-{p}_{0}{\}}^{T}$ and ${\varvec{R}}=[{r}_{ij}]$ is a coefficient matrix of the issue with elements ${r}_{1j}={e}^{-{\eta }_{j}h}$ ${r}_{2j}={B}_{j}{e}^{-{\eta }_{j}h}$, ${r}_{3j}={C}_{j}{\eta }_{j}{e}^{-{\eta }_{j}h}$, ${r}_{4j}=ik\lambda +(\lambda +2G){\eta }_{j}{B}_{j}$, ${r}_{5j}=G(ik{B}_{j}+{\eta }_{j})$, and ${r}_{6j}={C}_{j}$.

For the infinite seabed, the integral coefficients ${A}_{2}$, ${A}_{4}$ and ${A}_{6}$ must be zero to ensure the radiation conditions of displacement and pore pressure of seabed soil as $z\to -\infty$. Therefore, considering Eq. (13), the remaining coefficients ${A}_{j}$ ($j=1, 3, 5$) can be obtained as:

22 $${A}_{1}=\frac{{r}_{2}{p}_{0}}{{r}_{1}{C}_{3}-{r}_{2}{C}_{1}}, {A}_{3}=-\frac{{r}_{1}{p}_{0}}{{r}_{1}{C}_{3}-{r}_{2}{C}_{1}}, {A}_{5}=\frac{2{r}_{3}{p}_{0}}{{r}_{1}{C}_{3}-{r}_{2}{C}_{1}},$$

where $${r}_{1}=[{\eta }_{1}^{2}-\lambda {k}_{f}^{2}/(2G)]\left({k}_{t}^{2}+2{\eta }_{5}^{2}\right)-2{k}^{2}{\eta }_{1}{\eta }_{5}$$, $${r}_{2}=[{\eta }_{3}^{2}-\lambda {k}_{s}^{2}/(2G)]\left({k}_{t}^{2}+2{\eta }_{5}^{2}\right)-2{k}^{2}{\eta }_{3}{\eta }_{5}$$, $${r}_{3}=({\eta }_{3}{r}_{1}-{\eta }_{1}{r}_{2}){\eta }_{5}/({k}_{t}^{2}+2{\eta }_{5}^{2})$$.

After obtaining the expressions of integral coefficients, the stresses and excess pore pressure of the ocean sediment can be written as

23 $${\sigma }_{x}{\prime}=\sum_{j=1}^{6}[ik(\lambda +2G)+\lambda {B}_{j}{\eta }_{j}]{A}_{j}{e}^{{\eta }_{j}z}\left\{\left[\mathrm{cos}kx+{K}_{R}\mathrm{cos}\left(kx+\epsilon \right)\right]+i\left[\mathrm{sin}kx-{K}_{R}\mathrm{sin}\left(kx+\epsilon \right)\right]\right\}{e}^{-i\omega t},$$

24 $${\sigma }_{z}^{\mathrm{^{\prime}}}=\sum_{j=1}^{6}[i\lambda k+\left(\lambda +2G\right){B}_{j}{\eta }_{j}]{A}_{j}{e}^{{\eta }_{j}z}\left\{\left[\mathrm{cos}kx+{K}_{R}\mathrm{cos}\left(kx+\epsilon \right)\right]+i[\mathrm{sin}kx-{K}_{R}\mathrm{sin}(kx+\epsilon )]\right\}{e}^{-i\omega t},$$

25 $${\tau }_{xz}=G\sum_{j=1}^{6}(ik{B}_{j}+{\eta }_{j}){A}_{j}{e}^{{\eta }_{j}z}\left\{\left[\mathrm{cos}kx-{K}_{R}\mathrm{cos}\left(kx+\epsilon \right)\right]+i[\mathrm{sin}kx+{K}_{R}\mathrm{sin}(kx+\epsilon )]\right\}{e}^{-i\omega t},$$

26 $${p}_{f}=\sum_{j=1}^{6}{C}_{j}{A}_{j}{e}^{{\eta }_{j}z}\left\{\left[\mathrm{cos}kx+{K}_{R}\mathrm{cos}\left(kx+\epsilon \right)\right]+i\left[\mathrm{sin}kx-{K}_{R}\mathrm{sin}\left(kx+\epsilon \right)\right]\right\}{e}^{-i\omega t}.$$

The solutions derived can be degenerated to those for the dynamic response of seabed under progressive ocean waves (Wang et al.^24^) if the reflection of incident waves is omitted, i.e., letting ${K}_{R}=0$. Therefore, the solutions of Wang et al.^24^ are a special case of the present theory.

The present theory can also be easily degenerated to that provided by Tsai and Lee^7^ if the compressibility of solid and the influence of inertia forces are not considered, and the incident waves are assumed to be fully reflected with no phase difference between incident and reflected waves, i.e., letting ${\rho }_{s}={\rho }_{w}=0$, ${K}_{R}=1.0$, $\epsilon$ = 0, and ${K}_{s}\to \infty$. Therefore, the solutions of Tsai and Lee^7^ are also a special case of the proposed theory.

### Criterion of liquefaction potential

When ocean waves propagate above the seabed, there will be a cumulative increase in excess pore water pressure within the seabed. If the excess pore water pressure equals the total stress, the interparticle force between soil particles will be zero, and consequently the soil deposit is liquefied. There exist several quantitative criteria to judge the liquefaction of soil^9,25,40,41^. Among these criteria, the Jeng’s criterion^9^ considered the effects of horizontal stresses and excess pore water pressure and its physical meaning is clear. This criterion is expressed as^9^

27 $$-\frac{1}{3}\left({\gamma }_{s}-{\gamma }_{w}\right)\left(1+2{K}_{0}\right)z+{p}_{0}-{p}_{f}\le 0,$$

where ${K}_{0}$ is the coefficient of lateral earth pressure at rest, ${K}_{0}=\upsilon /(1-\upsilon )$; $\upsilon$ = Poisson’s ratio.

## Comparisons with existing results: verification

**Figure 2:**
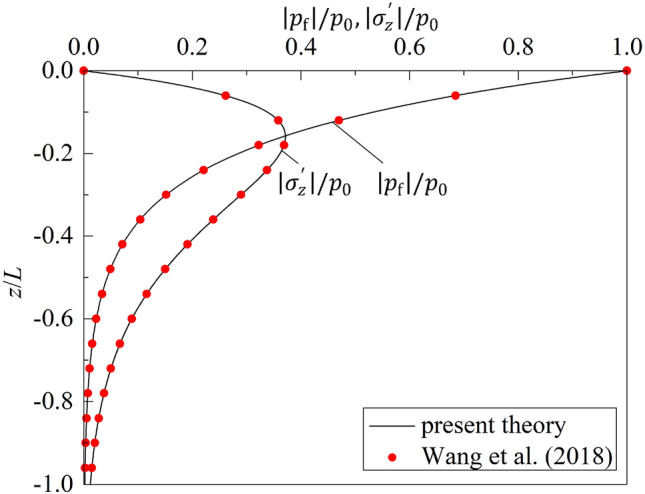
Comparison of degenerated present theory with that of Wang et al.^24^.

A series of theoretical investigations on the response and liquefaction of seabed induced by sanding or progressive ocean waves were carried out by Tsai and Wang^8,24^. In this section, these results will be compared with those obtained from the present theory. The parameters of waves and seabed soil used in the analysis are same as those used by Wang et al.^24^, which are listed in Table 1, unless otherwise stated. Firstly, the comparisons of effective vertical stress and excess pore water pressure for porous seabed of infinite thickness are carried out. As there is no wave reflection in Wang’s solution, ${K}_{\mathrm{R}}$ = 0.0 is chosen in the present theory. Figure 2 shows the comparisons of excess pore water pressure and effective vertical stress vs. $z/h$ calculated from the solutions of Wang et al.^24^ and the degenerated present theory. It can be clearly seen that the two results agree very well. The maximum relative difference of the present solution with those of Wang et al.^24^ is 0.13%.

**Table 1:** Wave and soil parameters used in the analysis.

Parameter	Value
Water wave	
Wave period, $T$ (s)	6.0
Wave height, $H$ (m)	2.0
Water depth, $d$ (m)	8.0
Wave length, $L$ (m)	45.2
Reflection coefficient,${K}_{\mathrm{R}}$	0.8
Phase lag, $\epsilon$ (°)	30.0
Seabed	
Seabed thickness, $h$ (m)	50.0 or infinity
Drained Poisson’s ratio,$\upsilon$	0.3
Porosity, $n$	0.4
Shear modulus, $G$ (Pa)	1.0 × 10^7^
Saturation, ${S}_{r}$ (%)	99.8
Mass density of grains, ${\rho }_{\mathrm{s}}$ (kg/m^3^)	2650
Mass density of fluid, ${\rho }_{w}$ (kg/m^3^)	1000
Coefficient of permeability, ${k}_{c}$ (m/s)	1.2 × 10^–3^
Bulk modulus of solid skeleton, ${K}_{\mathrm{s}}$ (Pa)	3.6 × 10^10^
Bulk modulus of air-free water, ${K}_{w}$ (Pa)	2.0 × 10^9^

**Figure 3:**
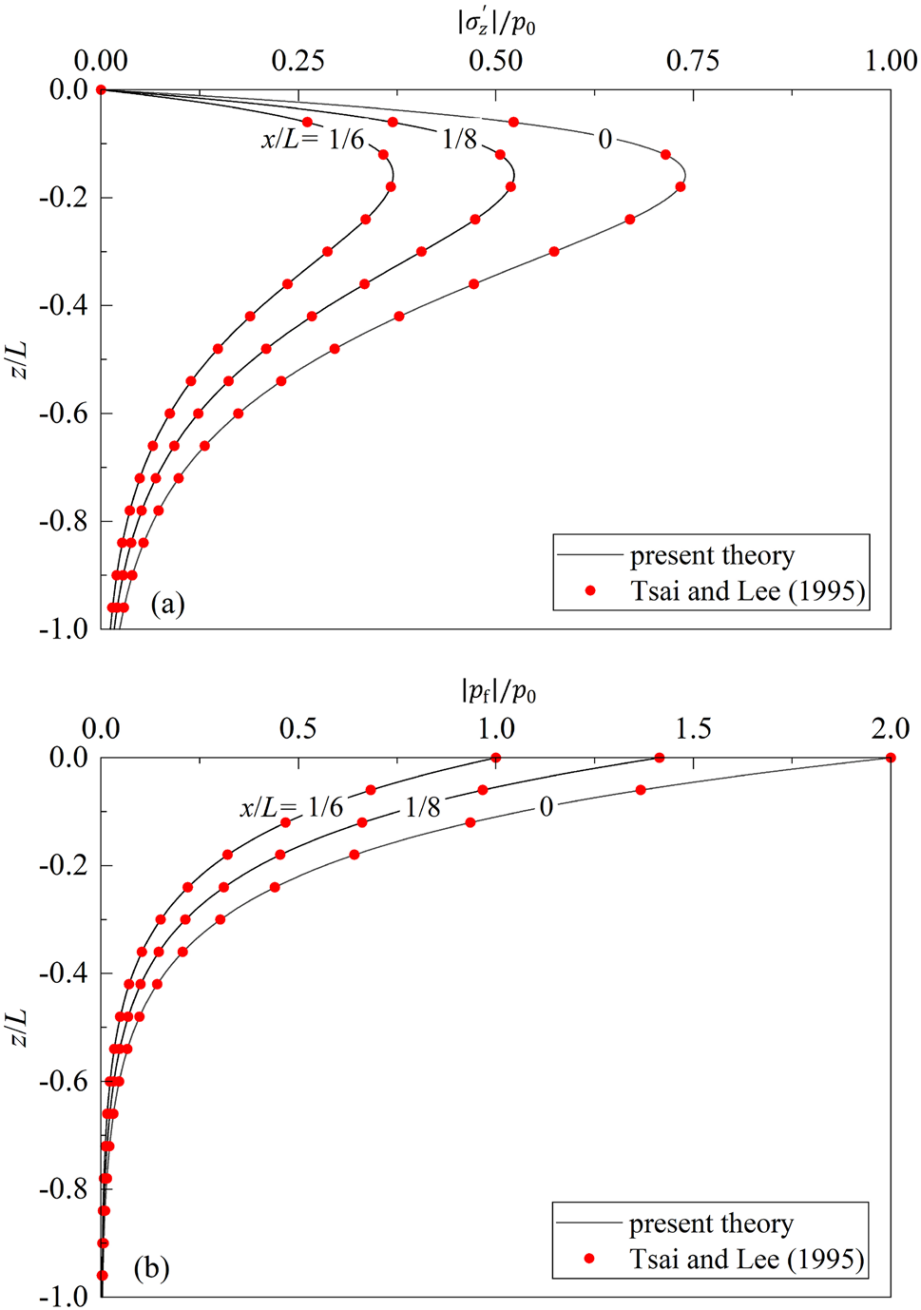
Comparison of degenerated present theory with that of Tsai and Lee^7^: (**a**) $\left|{\sigma }_{z}{\prime}\right|/{p}_{0}\sim z/h$; (**b**) $\left|{p}_{f}\right|/{p}_{0}\sim z/h$.

Then, the present theory is further compared with that of Tsai and Lee^8^. As the compressibility of solid skeleton, inertia effects of both solids and fluid, and the reflected wave is assumed to be fully reflected with no phase lag between incident and reflected waves in the Tsai’s solution^8^, ${\rho }_{\mathrm{s}}={\rho }_{\mathrm{w}}=0$, $K/{K}_{s}\ll 1$, ${K}_{R}$=1.0, and $\epsilon =0^\circ$ are taken in the degenerated present theory. The comparison results are shown in Fig. 3. One can note from the figure that the values of pore pressure and effective vertical stress vs. $z/h$ calculated from the two methods agree fairly well. The maximum relative differences of the present solution with those of Tsai and Lee^7^ is 0.15%.

## Parametric study and discussions

**Figure 4:**
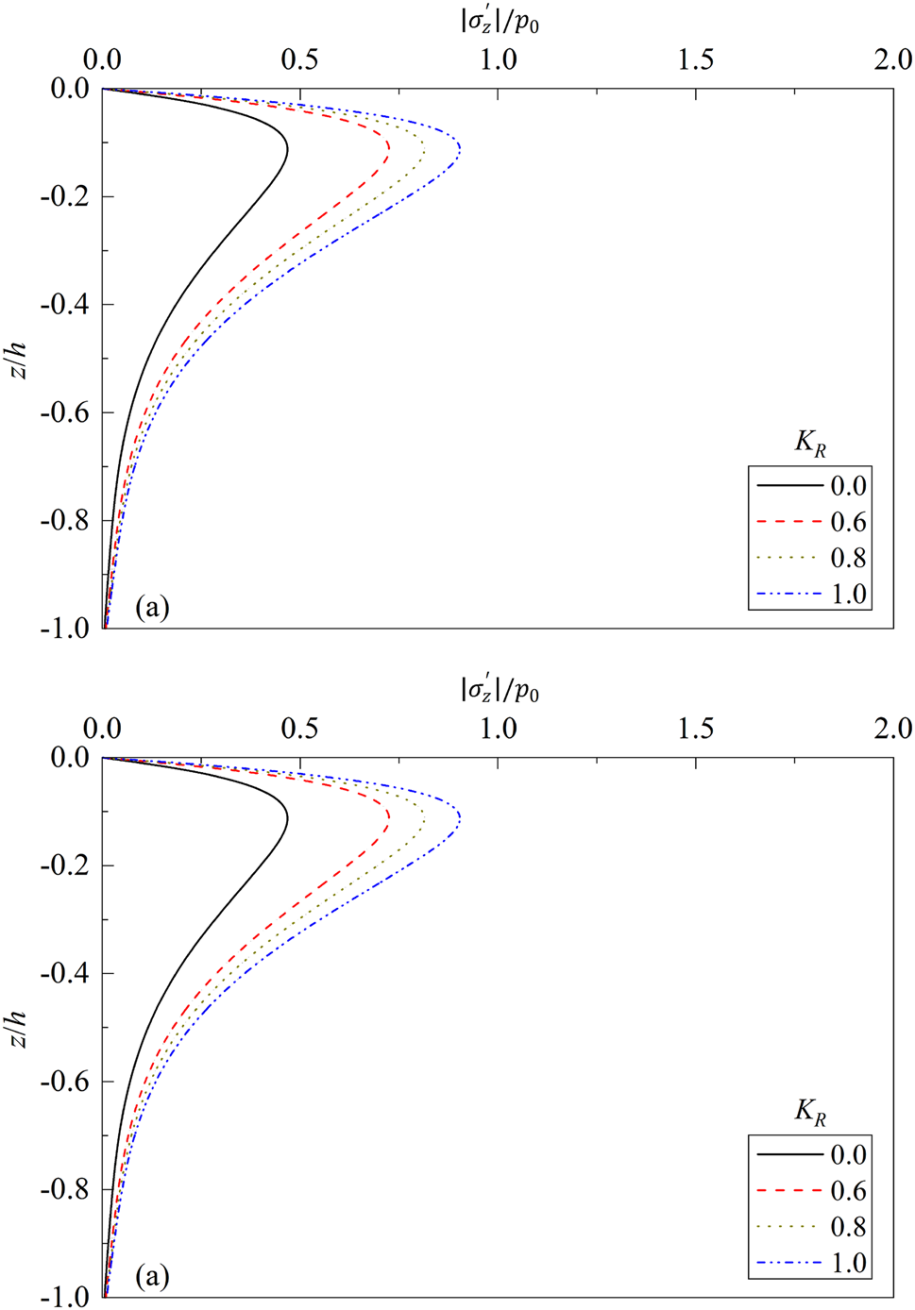
Partial standing-wave induced seabed response for different ${K}_{R}$: (**a**) $\left|{\sigma }_{z}{\prime}\right|/{p}_{0}\sim z/h$; (**b**) $\left|{p}_{f}\right|/{p}_{0}\sim z/h$.

**Figure 5:**
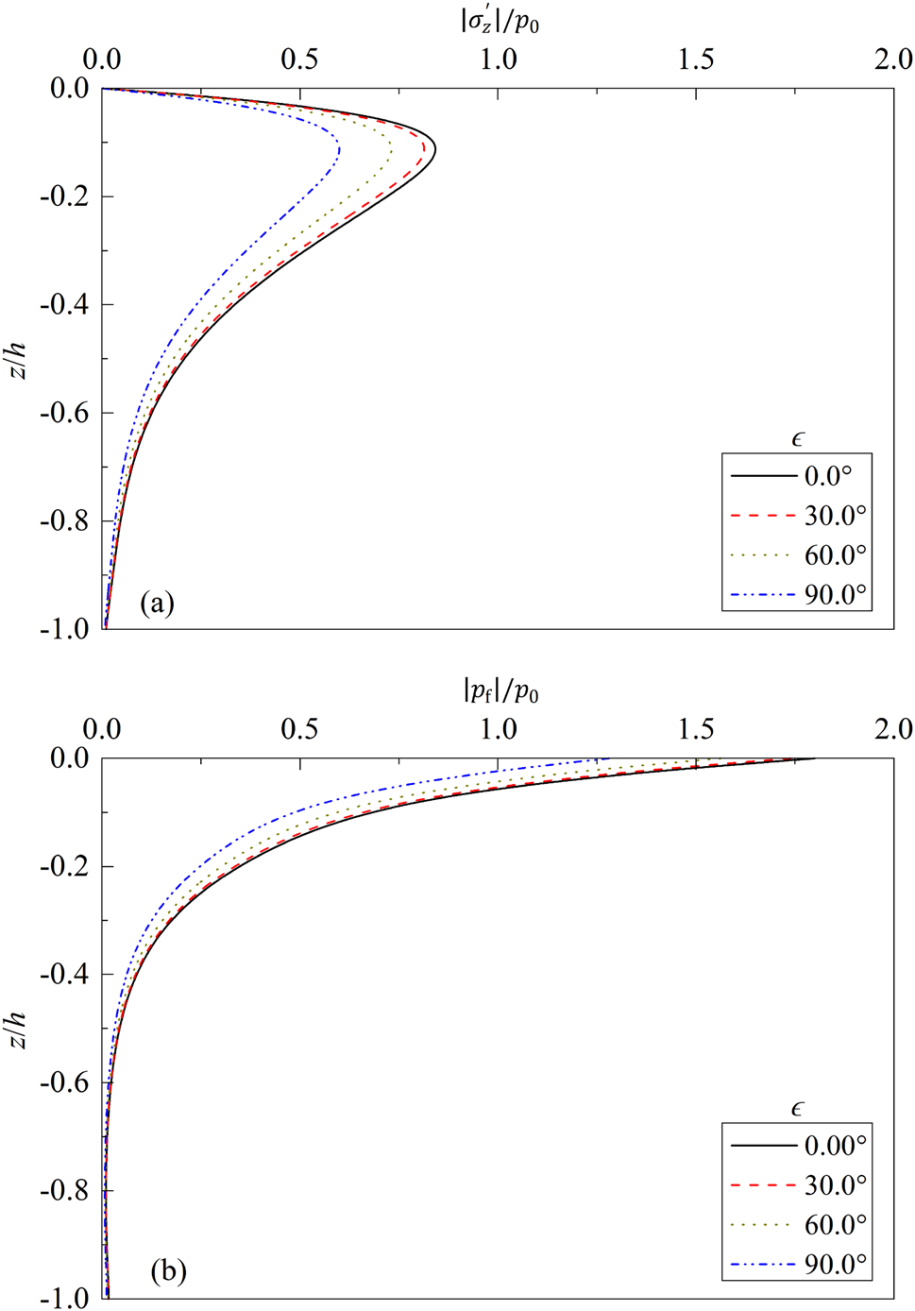
Partial standing-wave induced seabed response for different $\epsilon$: (**a**) $\left|{\sigma }_{z}{\prime}\right|/{p}_{0}\sim z/h$; (**b**) $\left|{p}_{f}\right|/{p}_{0}\sim z/h$.

**Figure 6:**
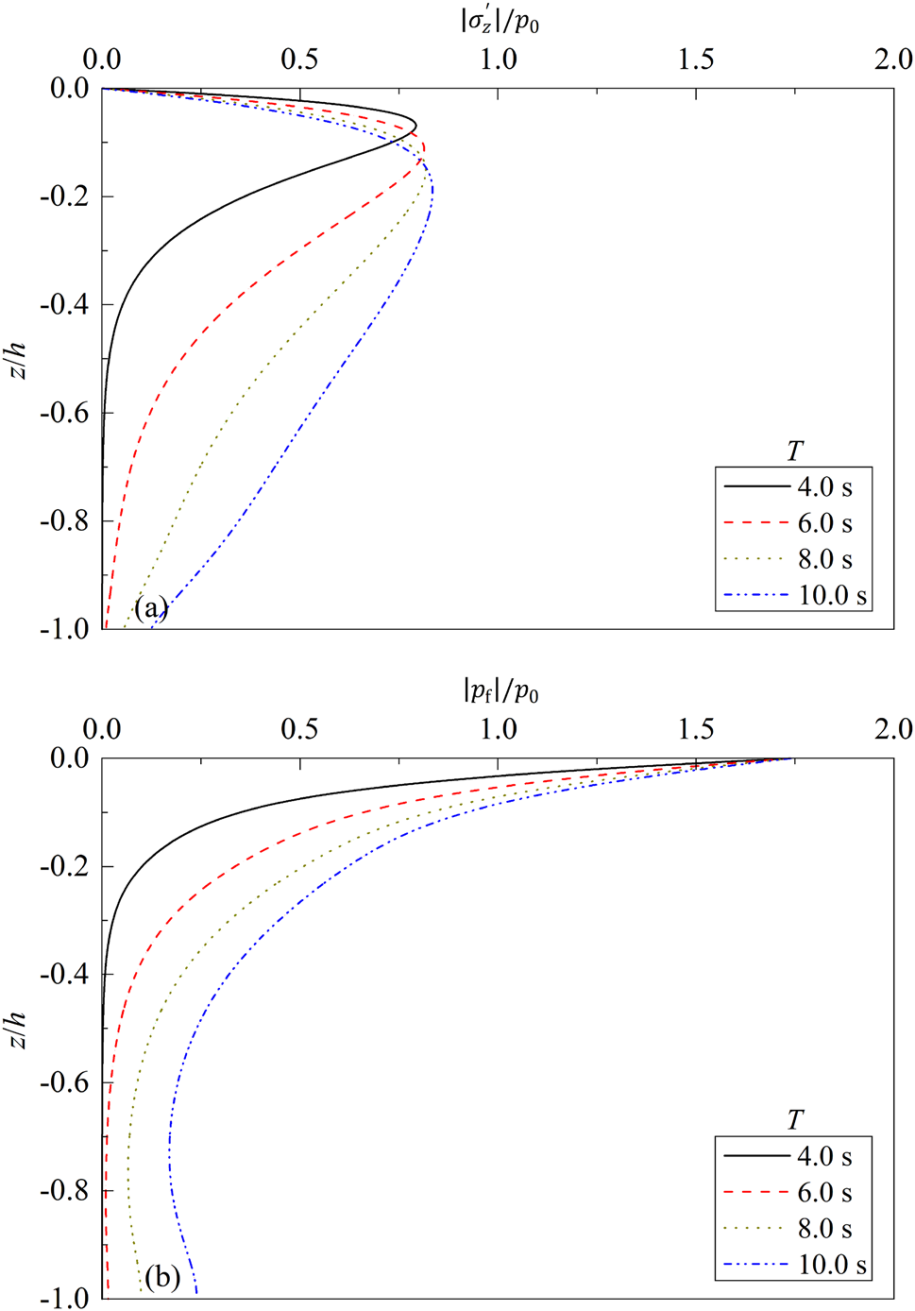
Partial standing-wave induced seabed response for different $T$: (**a**) $\left|{\sigma }_{z}{\prime}\right|/{p}_{0}\sim z/h$; (**b**) $\left|{p}_{f}\right|/{p}_{0}\sim z/h$.

**Figure 7:**
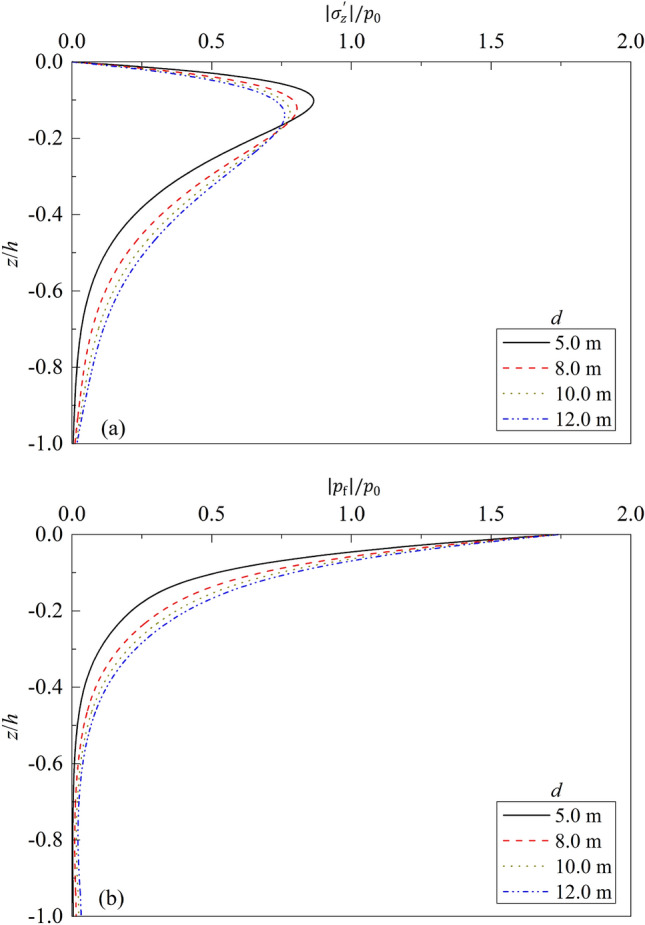
Partial standing-wave induced seabed response for different $d$: (**a**) $\left|{\sigma }_{z}{\prime}\right|/{p}_{0}\sim z/h$; (**b**) $\left|{p}_{f}\right|/{p}_{0}\sim z/h$.

**Figure 8:**
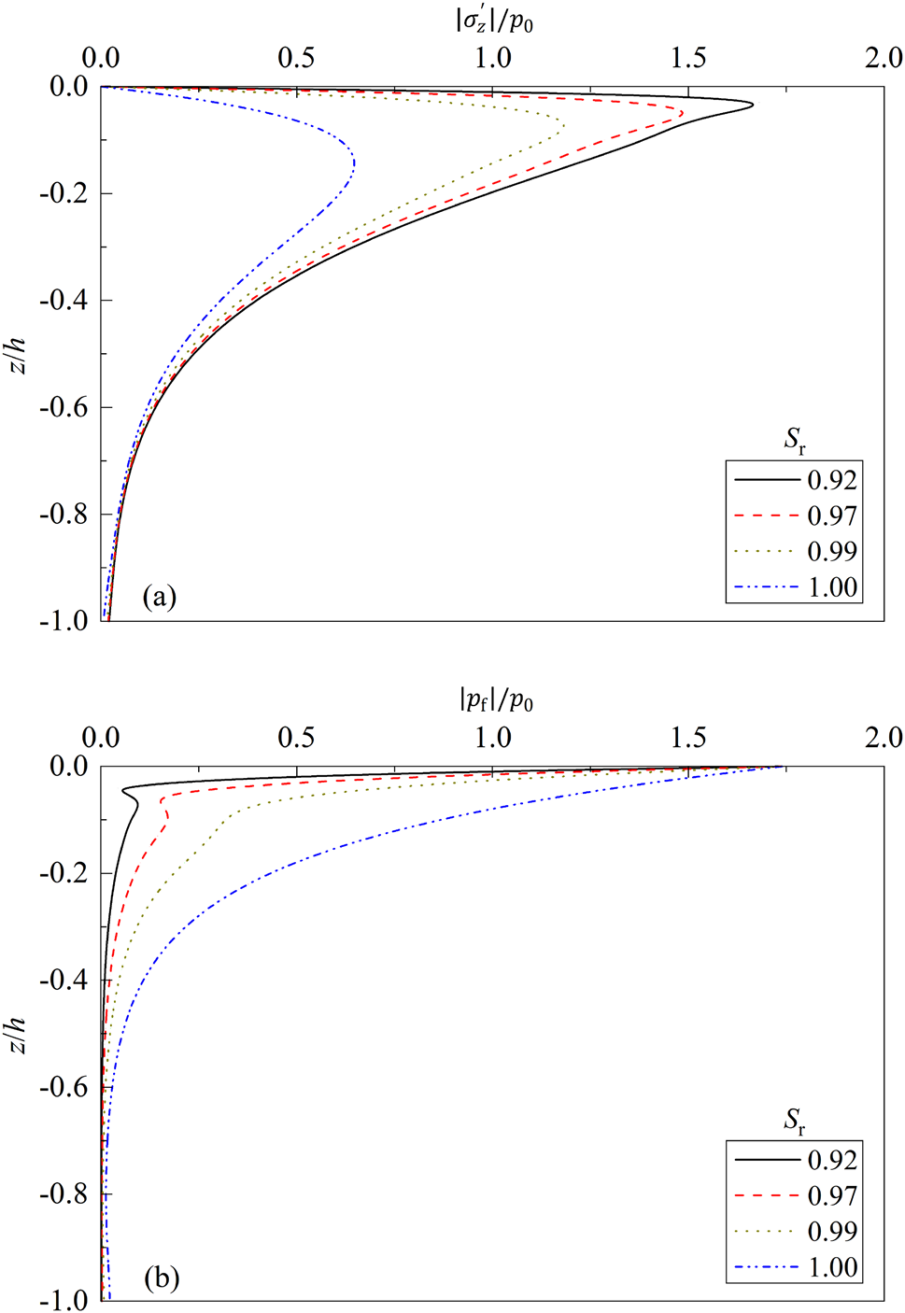
Partial standing-wave induced seabed response for different ${S}_{r}$: (**a**) $\left|{\sigma }_{z}{\prime}\right|/{p}_{0}\sim z/h$; (**b**) $\left|{p}_{f}\right|/{p}_{0}\sim z/h$.

**Figure 9:**
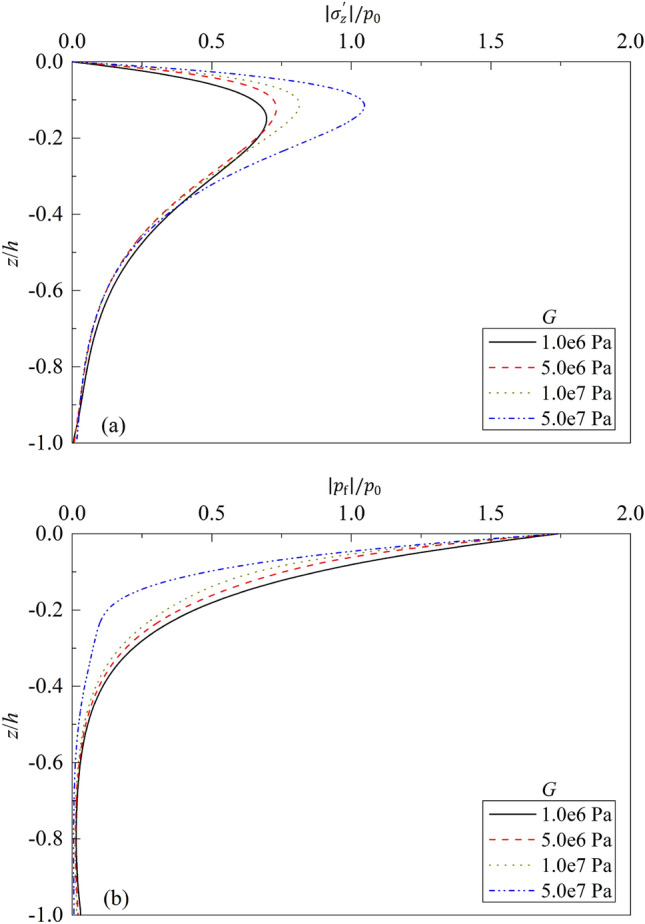
Partial standing-wave induced seabed response for different $G$: (**a**) $\left|{\sigma }_{z}{\prime}\right|/{p}_{0}\sim z/h$; (**b**) $\left|{p}_{f}\right|/{p}_{0}\sim z/h$.

**Figure 10:**
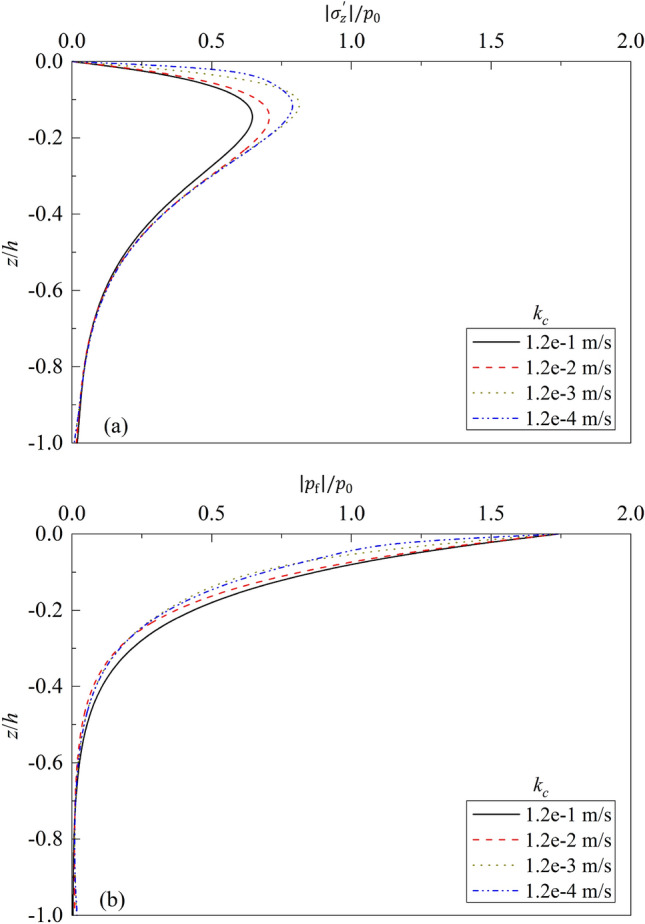
Partial standing-wave induced seabed response for different ${k}_{c}$: (**a**) $\left|{\sigma }_{z}{\prime}\right|/{p}_{0}\sim z/h$; (**b**) $\left|{p}_{f}\right|/{p}_{0}\sim z/h$.

**Figure 11:**
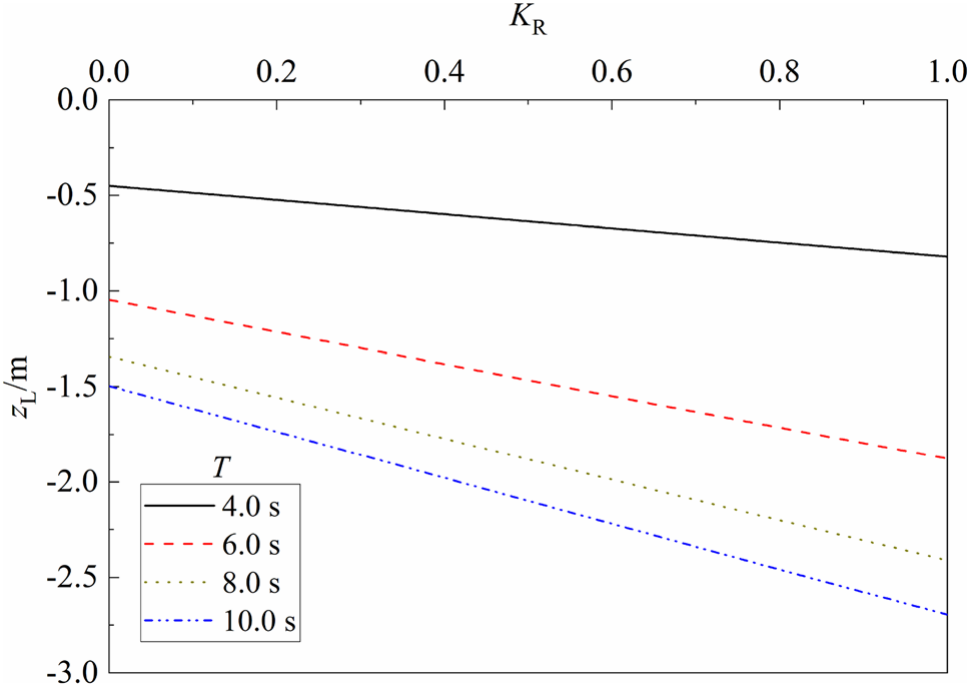
Variation of liquefaction depth vs. ${K}_{R}$ for different $T$.

**Figure 12:**
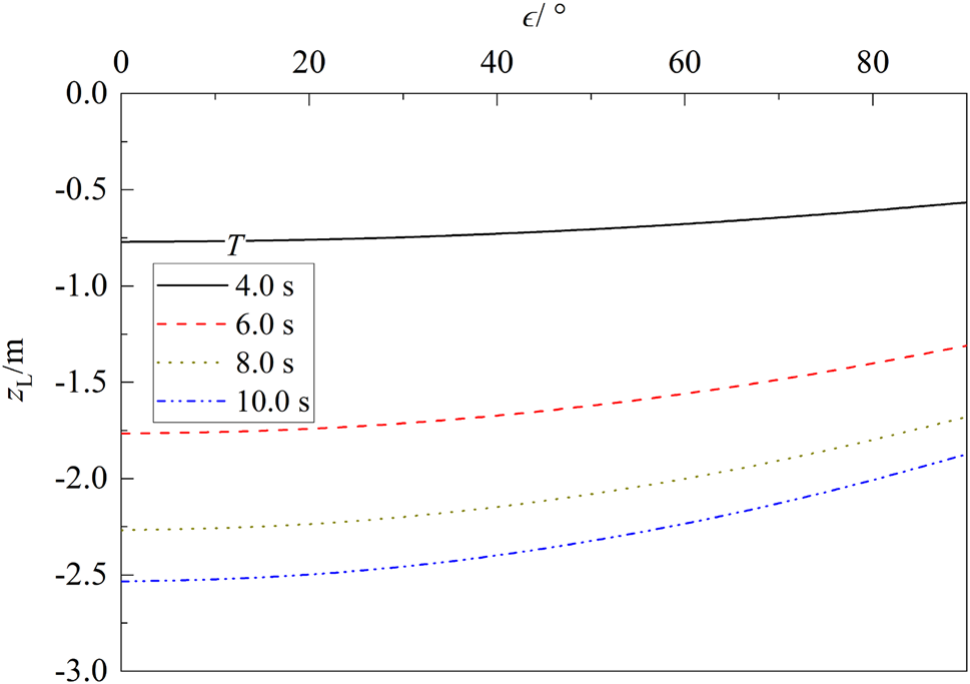
Variation of liquefaction depth vs. $\epsilon$ for different $T$.

**Figure 13:**
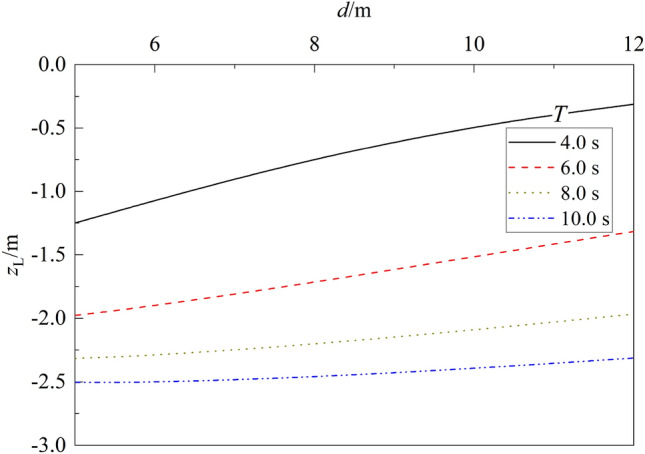
Variation of liquefaction depth vs. $d$ for different $T$.

**Figure 14:**
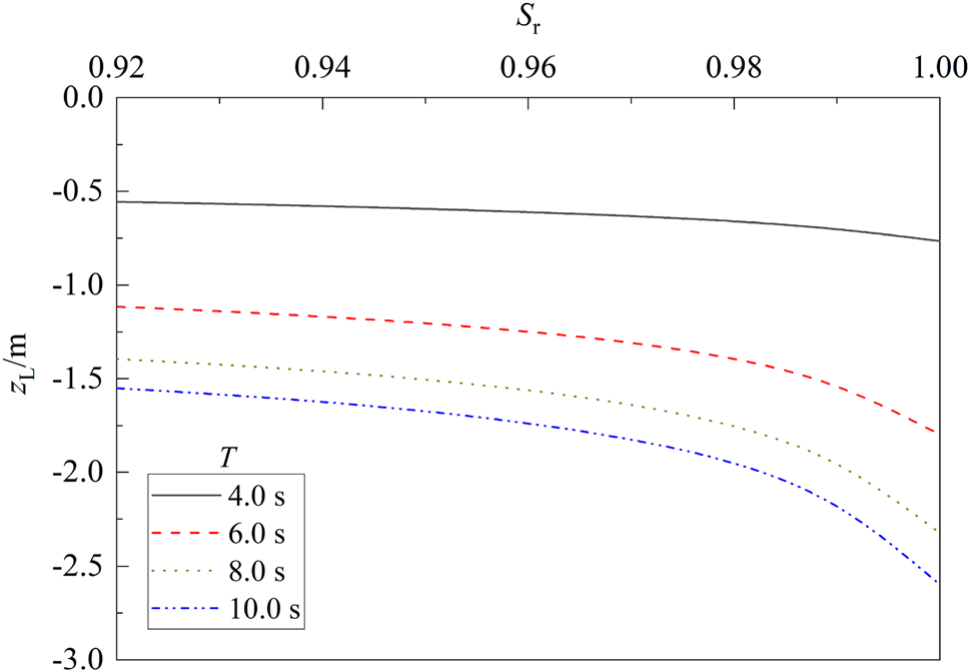
Variation of liquefaction depth vs. ${S}_{r}$ for different $T$.

**Figure 15:**
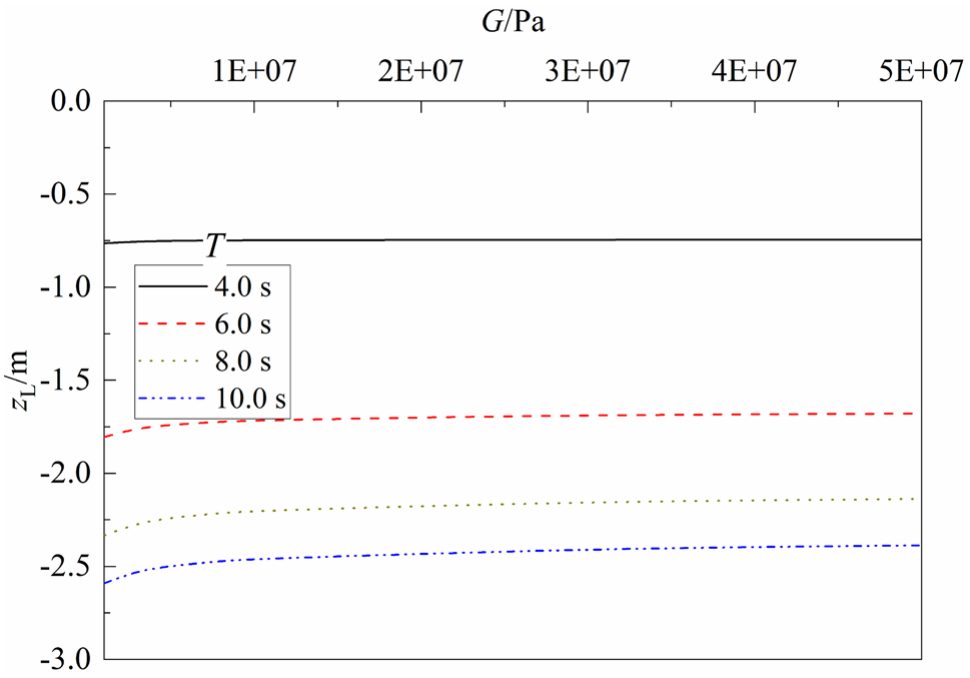
Variation of liquefaction depth vs. $G$ for different $T$.

**Figure 16:**
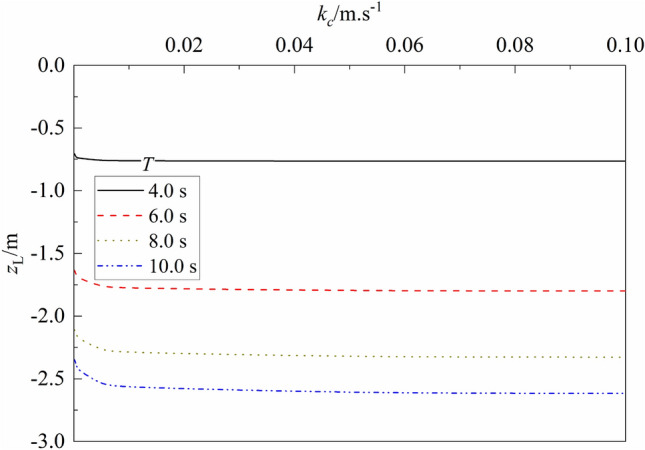
Variation of liquefaction depth vs. ${k}_{c}$ for different $T$.

In the parametric study sections, we refer to the relevant parameters of Wang et al.^24^ and Madsen^5^. Some selected results are given to investigate the influence of mechanical and physical parameters of ocean wave and soil on the dynamic response and liquefaction potential of porous seabed, especially the reflection coefficient and period of ocean waves, water depth, degree of saturation, shear modulus and permeability of marine sediment. The input data used in the analysis are same as those listed in Table 1, unless otherwise stated. The calculation results are listed in Figs. 4, 5, 6, 7, 8, 9, 10, 11, 12, 13, 14, 15 and 16.

### Dynamic response of porous seabed

To understand the partial standing wave-induced seabed response and liquefaction, this section gives the detailed discussions on the influence of different properties of partial standing waves and different parameters of seabed soil on the distribution of vertical stress, excess pore pressure and liquefaction potential of seabed.

Figure 4 shows the vertical distributions of dynamic response along seabed depth $z/h$ for different wave reflection coefficient. In the analysis, ${K}_{\mathrm{R}}$ = 0.0, 0.6, 0.8, and 1.0 are respectively used, where ${K}_{\mathrm{R}}=0.0$ indicates that there is no wave reflection, ${K}_{\mathrm{R}}=1.0$ indicates that the incident wave is fully reflected, and $0.0<{K}_{\mathrm{R}}<1.0$ indicates that the wave is partially reflected. The bigger the wave reflection coefficient is, the more intensive the standing wave consisting of incident and reflected waves will be. One can note from Fig. 4 that, for a given value of ${K}_{\mathrm{R}}$, the effective vertical stress increases gradually from 0.0, then begins to decrease gradually to 0.0 after reaching the peak value; while the excess pore pressure of seabed decreases gradually from maximum value to zero. For a given value of $z/h$, the excess pore pressure and effective normal stress increase gradually with the increase of ${K}_{\mathrm{R}}$. Compared with that for ${K}_{\mathrm{R}}=0.0$, the maximum value of vertical stress increases 54.89%, 73.94%, and 93.19% for ${K}_{\mathrm{R}}=0.6$, 0.8, and 1.0, respectively. This can be interpreted that the force caused by reflection waves applied on the water-seabed interface will increase accordingly as ${K}_{\mathrm{R}}$ increases. Therefore, in practical engineering constructions, some facilities, such as wave-absorbing devices, etc., can be installed to absorb part of the reflection waves when water waves encounter structures such as breakwaters during propagation, so as to reduce pore water pressure and normal stress in seabed soil, to improve the shear strength of soil, and thereafter to improve the stability of offshore structures and seabed foundations efficiently.

Figure 5 depicts the effect of phase lag $\epsilon$ on the effective vertical stress and excess pore pressure along seabed depth $z/h$. In the analysis, $\epsilon$ = 0.0°, 30.0°, 60.0°, and 90.0° are respectively chosen. It can be seen from Fig. 5 that for a given $\epsilon$, the effective vertical stress increases gradually from 0.0, then begins to decrease gradually to 0.0 after reaching the peak value; while the excess pore pressure of seabed decreases gradually from maximum value to zero. For a given $z/h$, the excess pore pressure and effective normal stress decrease gradually with the increase of $\epsilon$. Compared with that for $\epsilon$ = 0°, the maximum value of vertical stress decreases 3.37%, 13.22%, and 28.85% for $\epsilon$ = 30°, 60°, and 90°, respectively. This is due to the reason that the reflected waves are delayed due to the existence of phase lag $\epsilon$. The delay phenomena will be more obvious as $\epsilon$ increases. Therefore, in practical engineering projects, some facilities, such as jackstone and wing dams, can be installed in front of breakwaters to delay the arriving of reflection waves, so as to reduce pore water pressure and normal stress in seabed soil.

The effect of wave period $T$ on the dynamic response of a poroelastic seabed is given in Fig. 6. One can note that the change trends of the curves are similar to Figs. 4 and 5. At a given wave period $T$, the effective vertical stress increases gradually from 0, then begins to decrease after reaching the peak value as $z/h$ increases, but the excess pore water pressure ${p}_{f}$ decreases gradually as $z/h$ increases. At a given $z/h$, the values of effective vertical stress and excess pore water pressure in the lower layer of seabed increase intensively as $T$ increases. Under the same water depth and soil parameters, the wavelength will be longer for bigger wave period, consequently causing greater vertical stress and excess pore water pressure in the seabed soil.

Figure 7 depicts the effects of water depth $d$ on the dynamic response of a poroelastic seabed, where four values of $d$ are respectively considered, namely, $d$ = 2.0, 5.0, 8.0, and 10.0 m. One can note from Fig. 7 that for a given water depth $d$, the effective vertical stress increases along seabed depth $z/h$ gradually from 0, then begins to decrease after reaching the peak value, but the excess pore water pressure decreases gradually with the increase of $z/h$. At a given $z/h$, the value of effective vertical stress in the lower layer of seabed decreases as $d$ increases. The effective vertical stress in the lower layer of seabed increases with the increase of $d$, but the peak values almost the same for different water depth $d$. At a given $z/h$, the excess pore pressure increases with the increase of $d$.

The fluid compressibility ${K}_{f}$ is significantly connected with soil saturation. For two-phased saturated soil (${S}_{r}=1.0$), the bulk modulus of fluid is about 2.0e9 Pa. If there exists even a little gas in the fluid and the soil becomes unsaturated, then the compressibility of fluid will decrease heavily. Figure 8 depicts the variations of dynamic response of seabed vs. $z/h$ for different degree of soil saturation ${S}_{r}$. From Fig. 8, it can be seen that the dynamic response of upper soil layer is significantly affected by the degree of saturation ${S}_{r}$. For a given ${S}_{r}$, the vertical effective stress first increases along the seabed depth $z/h$, then begins to decrease after reaching the peak value. For a given $z/h$, the effective vertical stress decreases with the increase of ${S}_{r}$. Compared with that for ${S}_{r}$ = 0.92, the peak value of effective vertical stress decreases 11.23%, 29.55% and 61.50% for ${S}_{r}$ = 0.97, 0.99, and 1.0, respectively. Accordingly, the excess pore water pressure increases with the increase of ${S}_{r}$. This is due to the fact that the compressibility of air is much greater than that of pore water, resulting in lower wave pressure transmitted to the interior of the partially saturated seabed than that of the saturated one.

Figure 9 shows the distributions of the dynamic response of seabed vs. $z/h$ for different shear modulus $G$ of seabed soil. From the figure, it can be seen that the dynamic response of upper layer of soil is affected by $G$ significantly. Compared with that for $G$ = 1.0e6 Pa, the maximum value of effective vertical stress increases 5.03%, 16.94%, and 50.41% for $G$ = 5.0e6 Pa, 1.0e7 Pa, and 5.0e7 Pa, respectively. Consequently, the excess pore pressure decreases 13.34%, 25.42% and 52.84% for $G$ = 5.0e6 Pa, 1.0e7 Pa, and 5.0e7 Pa respectively. Therefore, it would be beneficial to the stability and liquefaction prevention of seabed by improving strength of the upper layer of seabed soil, such as cement mixing, replacing of soil with lower strength for better one, soil reinforcement, embedment of geotextiles, and so on.

Figure 10 depicts the influence of soil permeability ${k}_{c}$ on the dynamic response of poroelastic seabed. One can note from the figure that the permeability of soil has a significant influence on the vertical stress and excess pore water pressure within the upper layer of seabed soil, but has little influence on the dynamic response of lower layer. With the decrease of soil permeability, the value of effective vertical stress increases, and excess pore pressure decreases accordingly. Compared with that for ${k}_{c}$ = 0.12 m/s, the maximum value of ${\sigma }_{z}{\prime}$ increases 9.41%, 26.15%, and 22.27% for ${k}_{c}$ = 1.2e−2, 1.2e−3, and 1.2e−4 m/s respectively. Accordingly, the maximum value of ${p}_{f}$ decreases about 9.40%, 24.81% and 20.82% for ${k}_{c}$ = 1.2e−2, 1.2e−3, and 1.2e−4 m/s respectively. Therefore, in practical soil reinforcement, the methods, such as compaction, soil–cement mixing, the increase of clay content in sandy soil, can be used to decrease the permeability of upper layer of soil, of which good treatment effect can be achieved.

### Liquefaction potential of seabed

Figure 11 shows the influence of wave reflection coefficient ${K}_{R}$ and wave period $T$ on the liquefaction depth of porous seabed. One can note from the figure that for a given wave period $T$, the liquefaction depth ${z}_{L}$ increases gradually as ${K}_{R}$ increases. Compared with that for ${K}_{R}$ = 0.0, the liquefaction depth ${z}_{L}$ for fully reflected case (${K}_{R}$ = 1.0) respectively increases 82.49%, 79.28%, 79.62% and 80.12% for $T$ = 4.0, 6.0, 8.0 and 10.0 s. Compared with that for $T$ = 4.0 s, the maximum liquefaction depth ${z}_{L}$ increases about 132.94%, 198.65% and 232.89% for $T$ = 4.0, 6.0, 8.0 and 10.0 s, respectively.

Figure 12 depicts the influence of phase lag $\epsilon$ and wave period $T$ on the liquefaction depth of porous seabed. One can note from the figure that for a given wave period $T$, the liquefaction depth ${z}_{L}$ decreases gradually as $\epsilon$ increases. Compared with that for $\epsilon$ = 0.0°, the liquefaction depth ${z}_{L}$ for $\epsilon$ = 90.0° respectively decreases 26.58%, 25.88%, 25.96% and 26.08% for $T$ = 4.0, 6.0, 8.0 and 10.0 s. Compared with that for $T$ = 4.0 s, the maximum liquefaction depth ${z}_{L}$ increases about 131.55%, 197.02% and 231.38% for $T$ = 4.0, 6.0, 8.0 and 10.0 s, respectively.

The influence of water depth $d$ on the liquefaction depth ${z}_{L}$ of seabed is shown in Fig. 13. It can be seen from the figure that for a given wave period $T$, the liquefaction depth ${z}_{L}$ decreases gradually with the increase of $d$. Compared with that for $d$ = 5.0 m, the liquefaction depth ${z}_{L}$ for $d$ = 12.0 m decreases 74.98%, 33.43%, 15.08% and 7.66% for $T$ = 4.0s, 6.0s, 8.0s and 10.0 s respectively. The decrease of liquefaction depth is larger for smaller wave period $T$. This is due to that the bottom wave pressure applied on the surface of seabed decreases as water depth $d$ increases, resulting in the decrease of liquefaction depth accordingly.

Figure 14 depicts the variation of liquefaction depth vs. degree of saturation ${S}_{r}$ for different wave period. One can note from the figure that for a given wave period $T$, the liquefaction depth ${z}_{L}$ of seabed decreases as ${S}_{r}$ decreases. Compared with that for ${S}_{r}$ = 1.0, the liquefaction depth for ${S}_{r}$ = 0.92 decreases about 27.40%, 38.01%, 40.01%, and 40.33% for $T$ = 4.0, 6.0, 8.0, and 10.0 s, respectively. This is because that the permeability of soil is larger for smaller matrix suction and larger degree of saturation of seabed soil, causing the liquefaction depth to increase accordingly.

Figure 15 shows the variation of liquefaction depth ${z}_{L}$ versus shear modulus of seabed soil for different wave period. One can note from the figure that for a given wave period, the liquefaction depth of seabed decreases slightly as $G$ increases, but its influence is trivial and can be omitted. Compared with that for $G$ = 1.0e6 Pa, the maximum decrease of ${z}_{L}$ is 2.52%, 6.97%, 8.41%, and 7.85% for $T$ = 4.0, 6.0, 8.0, and 10.0 s, respectively. Figure 16 shows the variation of liquefaction depth ${z}_{L}$ versus permeability of seabed soil for different wave period $T$. It can be seen that for a given wave period, the liquefaction depth of seabed increases slightly as ${k}_{c}$ increases, but its influence is also trivial and can be omitted. This is because that the shear modulus and permeability of soil has little influence on the value of interparticle force. It can explain that silty soil and clayey soil with smaller shear modulus and lower permeability (compared with that of sandy soil) can also liquefy under applied loadings.

## Conclusions

A general analytical solution is developed to evaluate the dynamic response and liquefaction potential of poroelastic seabed induced by partial standing waves, where the effects of reflectivity of standing waves, and inertia terms of both solid skeleton and fluid relative to solid are considered. The explicit expressions of excess pore pressures, effective normal and shear stresses, and average displacements within the seabed soil of finite or infinite depth, have been developed analytically. Comparisons of the present theory with the existing ones show favorably good agreement. A detailed parametric study shows that the response and liquefaction potential of poroelastic seabed depends on the mechanical and physical properties of wave-seabed system, such as wave reflection coefficient, phase lag, and period of partial standing wave, water depth, degree of saturation, permeability and shear modulus of seabed soil, etc. Compared with that induced only by incident waves, the amplitude of soil response induced by fully reflected standing waves may reach two-fold. For different water depth, wave period, degree of saturation and shear modulus of soil, the response and liquefaction potential of seabed induced by partial standing wave is obviously different.

However, there are still some deficiencies in this study. This study only uses the general theory established by Biot to describe the dynamic behavior of poroelastic saturated media, and there is still a lack of necessary research on the influence of gas phase in the seabed. In the future, more realistic unsaturated media or gas-bearing soil media should be considered for research. In addition, the linear wave theory used in this study is more suitable for sandy seabed with higher permeability. However, when considering more complex soil deformation and liquefaction, a more professional elastic–plastic soil model should be adopted. These restrictions were added to the revised manuscript.

## Data Availability

Data underlying the results presented in this paper can be obtained from the corresponding author upon reasonable request.
